# T81. LONG-TERM COURSE OF COGNITIVE PERFORMANCE IN PATIENTS WITH CHRONIC SCHIZOPHRENIA

**DOI:** 10.1093/schbul/sby016.357

**Published:** 2018-04-01

**Authors:** Thaís Martins, Thalita Fernandes, Diego Mendes, Gustavo Mustafé, Luis Fernando Pegoraro, Clarissa Dantas

**Affiliations:** Univeristy of Campinas

## Abstract

**Background:**

Cognitive deficits are prevalent among patients with schizophrenia and are robustly associated with functioning and outcome. Although cognitive deficits are known to be present at the prodromal phase and to worsen at the onset of the disease, the long-term course of cognitive impairments are less well established. Many studies have focused on first episode psychosis with relatively short lengths of follow-up. Thus, the aim of this study is to investigate changes in cognitive performance of chronic schizophrenia patients in a variety of neurocognitive tests over a seven-year test-retest period.

**Methods:**

We will contact 85 patients with schizophrenia (as defined by the DSM-IV-TR), considered clinically stable in the previous year, who participated in a study about the deficit syndrome of schizophrenia carried out in 2009 and 2010. Back then, they were recruited in two sites: an outpatient psychiatric service of a university general hospital (49 patients) and a community-based mental health service for patients with severe mental illness (36 patients), both in Campinas, Sao Paulo, Brazil. Patients will be assessed with the same instruments adopted in the first study: a questionnaire for clinical and demographic information; SAPS, SANS, Calgary Depression Scale and a battery of neurocognitive tests comprising: Digit Span Forward (DSF), Digit Span Backward (DSB), Rey Complex Figure Copy (RCFC), Rey Complex Figure Memory (RCFM), Digit symbol-coding (DSC), Picture Completion (PC), Matrix Reasoning (MR), Vocabulary (V), Trail Test A (TTA), Trail Test B (TTB), Phonological Fluency (PF) and Semantic Fluency (SF). To test differences in neurocognitive performance, and in symptoms severity at base and at follow-up we used the Wilcoxon Test.

**Results:**

We present in this poster partial results. Among the 20 reassessed patients the mean age at baseline was 36.9 ± 8.9 years, mean duration of mental illness was 16 ± 10.1 years, 75% were men. They had, in mean, 10.7 ± 3.3 years of education, only 20% had any work activity, and 15% were married. Mean length of test-retest interval was 6.9 years (minimum 6 and maximum 7.7). At follow-up, 4 patients had improved their education, but only 3 (15%) had any work activity. Up to now 19 patients completed the cognitive reassessment. Severity of positive and of depressive symptoms was low at base line (mean score on SAPS 5.5 ± 4.8; mean score on Calgary 1.5 ± 1.9) and remained low at follow-up (SAPS 6.2 ± 4.4, Calgary 2.2 ± 2.2), with no significant change. Patients, as a group, had moderate negative symptoms were at baseline (mean SANS score 10.5 ± 6.9) and had their negative symptoms worsened at follow-up (SANS 14.8 ± 7.1), p=0.005. Patients had a worse performance at follow-up in 4 out of 12 tests: DSF (3.8 ± 1.5 at follow-up versus 10.1 ± 2.8 at baseline, p < 0.000), DSB (3.4 ± 1.9 at follow-up versus 4.3 ± 2.2, p=0.005), RCFC (14.8 ± 9.4 versus 30.2 ± 8.6, p < 0.000) and RCFM (5.9 ± 6.5 versus 13.9 ± 9.8, p < 0.000). In the remaining 8 tests: DSC, PC, MR, V, TTA, TTB, PF and SF, there were no significant differences in performance between baseline and follow-up assessments.

**Discussion:**

Our preliminary results are derived from a small sample. Although we cannot draw definite conclusions, we identified different patterns of longitudinal course for different cognitive domains with attention, shot-term memory, working memory, visual-spatial ability and executive functions presenting a decline over time; whereas other domains, such as visual memory, visual perception, learning memory, verbal comprehension, motor function, remaining stable in patient through patients’ 4th and 5th decades of life.

